# Medication use in Northern Ireland in early pregnancy: Agreement between maternal self-report and prescribing records

**DOI:** 10.23889/ijpds.v8i2.2262

**Published:** 2023-09-14

**Authors:** Joanne Given, Helen Dolk, Maria Loane

**Affiliations:** 1 Ulster University, Belfast, United Kingdom

## Objectives

To assess the agreement between medication data reported by the mother at the pregnancy booking interview and recorded by midwives in the Northern Ireland Maternity System (NIMATS) compared to dispensed prescription data recorded in the Enhanced Prescribing Database (EPD).

## Methods

As part of a larger cross-sectional study investigating the prevalence of prescriptions dispensed between the last menstrual period (LMP) and the first antenatal visit 2011-2016, this sub-study assessed the agreement between self-reported medication use recorded in NIMATS and EPD compared to NIMATS, 2015-2016. Sensitivity and specificity compared to EPD were also calculated. This shorter time period was used due to underreporting of non-supplement medications before 2015 in NIMATS.

## Results

Between 2015-2016, there were 34,432 pregnancies to 34,096 women. In NIMATS 96.8% of women reported taking medications during early pregnancy compared to 64.3% of women having prescriptions dispensed in EPD. Folic acid (low and high-dose) was the most common medication recorded in both NIMATS (57.2%) and EPD (26.2%). Antibiotics, analgesics, antacids and anticoagulants were more commonly recorded in NIMATS but all other non-supplement medications were more common in EPD. Agreement between NIMATS and EPD ranged from 2.6% for vitamins to 87.8% for insulin. Sensitivity ranged from 2.5% for cardiac medications to 84.3% for anticoagulants. Specificity ranged from 47.6% for antibiotics to 100% for cardiac and antiviral medications. Folic acid (low and high-dose), vitamins, antibiotics and analgesics were the only medications with specificity less than 90%.

## Conclusion

Over-the-counter medications such as low-dose folic acid, antacids and analgesics were recorded more at the maternity booking interview (NIMATS), while medications requiring prescriptions were recorded more in EPD. This is important information for determining which data source to use in future studies assessing medication use.

